# Nitric-acid

**Published:** 1896-12

**Authors:** 


### HOMŒOPATHIC PHYSICIAN,

A MONTHLY JOURNAL OF

HOMŒOPATHIC MATERIA MEDICA AND CLINICAL MEDICINE.

"If our school ever gives up the strict inductive method of Hahnemann, we are lost, and deserve only to be mentioned as a caricature in the history of medicine."—CONSTANTINE HERING.

Vol. XVI. DECEMBER, 1896. No. 12.

### EDITORIAL.

NITRIC-ACID.—The stitching pains in and between the shoulder-blades, the pain in the small of the back from taking cold and the swelling of the glands of the neck and axilla, are indications for both Calcarea and Nitric-acid.

Nitric-acid has falling to sleep of the hands, especially in the morning in bed. Pain as from tension in right hip-joint. According to Dr. Malcolm Macfarlan, one of the great characteristics of Nitric-acid is soreness. There is soreness of the corners of the mouth; soreness of the inside of the nostrils; soreness of the shins. The shins are so sore the patient wishes to cover them with wet cloths.

Nitric-acid has a great characteristic of sticking pains or stitches, as if from needles or splinters. In this it is similar to Hepar. There are stitches in the heels when stepping; the patient feels as if stepping on needles.

Nitrie-acid has offensive foot-sweat, and so also has Silicea. The Nitric-acid foot-sweat causes soreness between the toes. Those who wish to get a comprehensive set of indications for foot-sweat should consult THE HOMŒOPATHIC PHYSICIAN for June, 1894, where will be found an elaborate repertory of foot-sweat prepared by Dr. Olin M. Drake. A few reprints of this repertory are still to be had in this office.

The editor has been repeatedly appealed to by his patients to stop the intolerable odor of the feet in valued servants, who, in consequence of this objection, were felt by the family to be unendurable. A careful study of the symptoms has resulted in the selection of a remedy that has entirely cured the trouble. Most of the cases seemed to respond best to Silicea, and rarely has there been a failure to relieve.

Nitric-acid is the remedy for bunions with swelling and with stinging pains. Nitric-acid, said Dr. Lippe, is almost a specific for bunions.

Nitric-acid has sore pains of internal organs. Emaciation of the upper extremities is an indication for Nitric-acid. Nitric-acid is a great remedy for syphilis after the abuse of Mercury. It also follows Mercury, homœopathically given, in syphilitic cases.

It is like Thuja, a great remedy for sycotic condylomata.

During sleep there is bleeding of the nose, and this is similar to Mercury. Coughing and sneezing during sleep is another indication for Nitric-acid.

Continuous chilliness is a symptom of Nitric-acid, and also reminds us of Pulsatilla.

Nitric-acid has perspiration at night on the side on which he lies, whilst Silicea has perspiration on the side on which he does *not* lie. Symptoms like this are liable to be overlooked, yet if kept well in mind are invaluable in selecting the remedy. Chill, heat, and sweat following one another suggest China and Pulsatilla as well as Nitric-acid.

Nitric-acid and Apis have nettle rash, with burning, stinging pains.

Nitric-acid has large blood boils like Arnica. The great characteristic of Nitric-acid in boils is the pain like *splinters*.

Painless swelling of the glands is the indication for Silicea. Nitric-acid has swelling of the glands with pain as if from splinters.

Hahnemann's key-note for Nitric-acid is that the drug is suitable after alkalies for lean persons with dark complexion, black hair and eyes.

Nitric-acid has aggravation after breakfast, in warm air, after lying down, after perspiration, and in the erect posture. Sulphur also has aggravation in the erect position. In fact, in some conditions the Sulphur patient is totally unable to stand erect, but must bend over.

Nitric-acid has amelioration from drawing clothing tight around the waist. This is the reverse of a number of remedies.

Dr. Guernsey's key-notes for Nitric-acid are as follow:

Leucorrhœa of mucus, which can be drawn out. It may be flesh-colored, greenish, cherry red, and fetid. Violent pressing downward, as if everything were coming out of the vulva, with pain in the small of the back, through the hips and down the thighs. Very painful stools with profuse discharge of blood, the pain lasting a long time and exhausting her. The urine is very strong, like horse urine. Sleeps badly the latter part of the night. Suitable for cases that are suffering from mercurial poisoning. Stitches in the vagina from without inwards when walking in the open air. Tumors, with much itching and stitching pains. Itching of the parts when walking, or otherwise irritating them, when they feel very sore. Pricking pains prevail. Violent itching of the vulva, always toward evening.

Swelling and burning itching of one side of the vulva and vagina. Dry-burning heat of the vulva. The urine is very offensive. Patient always worse after twelve o'clock at night. Violent cramp-like pains, as if the abdomen would burst with constant eructations. Hard knots in the mammary glands of mercurialized women. Constant eructations during the menses. Menses too early and too profuse. Metrorrhagia after confinement or miscarriage. Much nausea and gastric trouble, relieved by moving about or riding in a carriage. Constant nausea, with heat in the stomach extending to the throat. Fat food causes nausea and acidity. Hard, difficult, scanty stool. On going to stool pain in rectum, as if something were being torn away, or else twitchings in the rectum and spasmodic contractions of anus for many hours afterward. Smarting more in the rectum than in the anus immediately after stool and continuing many hours afterward. Sometimes prolapse of rectum and discharge of blood. Pain before and after stool as from a fissure of anus.

Much swelling of the internal ear. It is nearly closed. There is much pain in it. Much restlessness after midnight.

